# High expression of prolactin receptor is associated with cell survival in cervical cancer cells

**DOI:** 10.1186/1475-2867-13-103

**Published:** 2013-10-22

**Authors:** Edgar I Lopez-Pulido, José F Muñoz-Valle, Susana Del Toro-Arreola, Luis F Jave-Suárez, Miriam R Bueno-Topete, Ciro Estrada-Chávez, Ana Laura Pereira-Suárez

**Affiliations:** 1 Doctorado en Ciencias Biomédicas, Centro Universitario de Ciencias de la Salud, Universidad de Guadalajara, Guadalajara, Jalisco, México; 2 Grupo de Inmunogenética Funcional, Departamento de Biología Molecular y Genómica, Centro Universitario de Ciencias de la Salud, Universidad de Guadalajara, Guadalajara, Jalisco, México; 3 Laboratorio de Inmunología, Departamento de Fisiología, Centro Universitario de, Ciencias de la Salud, Universidad de Guadalajara, Guadalajara, México; 4 División de Inmunología,Centro Médico Nacional de Occidente (CMNO), Instituto Mexicano del Seguro Social(IMSS), Guadalajara, Jalisco, México; 5 Instituto de Enfermedades Crónico-Degenerativas, Departamento de Biología Molecular y, Genómica, Centro Universitario de Ciencias de la Salud, Universidad de Guadalajara, Guadalajara, Jalisco, México; 6 Unidad de Biotecnología Médica y Farmacéutica, Centro de Investigación y Asistencia en, Tecnología y Diseño del Estado de Jalisco AC, Guadalajara, Jalisco, México

**Keywords:** Cervical cancer, PRLR, PRL, Proliferation, Apoptosis

## Abstract

**Background:**

The altered expression of prolactin (PRL) and its receptor (PRLR) has been implicated in breast and other types of cancer. There are few studies that have focused on the analysis of PRL/PRLR in cervical cancer where the development of neoplastic lesions is influenced by the variation of the hormonal status. The aim of this study was to evaluate the expression of PRL/PRLR and the effect of PRL treatment on cell proliferation and apoptosis in cervical cancer cell lines.

**Results:**

High expression of multiple PRLR forms and PRLvariants of 60–80 kDa were observed in cervical cancer cell lines compared with non-tumorigenic keratinocytes evaluated by Western blot, immunofluorecence and real time PCR. Treatment with PRL (200 ng/ml) increased cell proliferation in HeLa cells determined by the MTT assay at day 3 and after 1 day a protective effect against etoposide induced apoptosis in HeLa, SiHa and C-33A cervical cancer cell lines analyzed by the TUNEL assay.

**Conclusions:**

Our data suggests that PRL/PRLR signaling could act as an important survival factor for cervical cancer. The use of an effective PRL antagonist may provide a better therapeutic intervention in cervical cancer.

## Background

Prolactin (PRL) is a pituitary polypeptide hormone with multiple biological actions which include proliferation and differentiation of mammary gland cells, beginning and maintenance of lactation, immunoregulation, osmoregulation, behavior and reproduction [1]. The role of PRL in tumorigenesis was suggested some decades ago in breast cancer, mainly in animal-based research [2]. However, the relevance of extrapolating these results to humans has always been questioned. Epidemiological studies performed during the 80s and 90s were unable to reach unified conclusions from correlations between circulating PRL levels and risk for breast cancer [3,4]. Also, clinical studies report that reducing circulating PRL levels did not improve the condition of advanced breast cancer patients [5]. This controversial view of PRL in human cancer has been considerably modified in the past 10 years. Now, there is evidence that high circulating PRL levels are considered a risk factor in breast cancer [6,7] and in other reproductive cancers such as endometrial, ovarian and prostate [8,9]. In addition to circulating PRL, there is clear evidence that several human tissues also express PRL like the mammary gland, prostate, skin, decidua, brain, some immune cells, adipocytes, and several others [10]. The biological effects of PRL are mediated by its interaction with the PRL receptor (PRLR). As PRLR is also expressed in these tissues, co-expression of both partners suggests the existence of an autocrine–paracrine loop of action. In recent years, reports supporting the tumor growth potency of local PRL in humans are emerging [11-14]. Alternative strategies involving PRLR neutralization and PRL antagonists opened new areas of research in this field.

PRLR belongs to the superfamily of hematopoietic cytokine receptors. Binding of PRL activates several signaling pathways, which include the Janus kinase-Signal transducer and activator of transcription (Jak-Stat), the Mitogen-activated protein kinases (MAPK), and the phosphoinositide 3 kinase (PI3K). Activation of these cascades results in endpoints such as differentiation, proliferation, survival, and secretion [15,16]. There are several prolactin receptor isoforms identified in humans, including the long form , an intermediate form, two short forms and soluble receptor isoforms, all of them generated through mRNA splicing [17-19]. Each of these forms has the same extra-cellular sequence, but differs in the intra-cellular signaling. The effects of PRL are dependent on the expressed PRLR form(s) of PRLR expression; the long and intermediate forms have been associated with increased cell proliferation or anti-apoptotic effects while the short and soluble forms have been described as being dominant negative [20]. Another mechanism potentially participating in local amplification of PRLR signaling in tumor contexts has recently emerged, and involves gain-of-function of PRLR variants.

Cervical cancer is a leading cause of morbidity and mortality among women worldwide, especially in developing countries [21]. Infection with oncogenic types of Human Papillomavirus (HPV) is an important factor in the development of cervical cancer [22]. Despite the evidence that HPV is strongly implicated as the causative agent of cervical cancer, this infection alone is not sufficient for tumor development. In addition, the immune system, as well as microbial, chemical [23,24] and hormonal cofactors play a role in the development of neoplastic lesions in the uterine cervix. Indeed, the variation of the hormonal status depending on age, pregnancy or contraceptive use, has been shown to influence the development of cervical cancers [25-27]. PRL expression in tissues and serum has been found elevated in patients with cervical cancer [28,29] suggesting a possible participation in the development or progression of the disease. Hence, PRLR expression in cervical cancer has not been well documented and the roles of PRL and PRLR in tumor development are still unknown. In the present study, we determined the expression levels of PRL and PRLR and the effect of PRL treatment on cell proliferation and apoptosis in HeLa, SiHa and C-33A cervical cancer cell lines. In addition, we antagonized any possible effect of the locally produced PRL using specific blocking antibodies against PRL and PRLR.

## Methods

### Standard culture conditions

Cervical cancer cell lines (HeLa, SiHa, and C-33 A), as well as two human breast cancer cell lines (MCF-7, T-47D) and non-tumorigenic human keratinocytes (HaCaT) were obtained from the American Type Culture Collection (Rockville, MD); cells were cultured in a water-jacketed incubator at 37°C under an atmosphere of 95% air and 5% CO2 in RPMI 1640 or DMEM medium supplemented with 10% fetal bovine serum (FBS), L-glutamine (2 mM), penicillin (100 U/ml), streptomycin (100 μg/ml). Cell medium, FBS and antibiotics were obtained from Gibco (Carlsbad, CA).

### Western blotting

Cells were seeded into 6-well plates and grown to 80% confluence in growth medium. Proteins were extracted from cell lines with 300 μl of RIPA buffer (50 mM Tris, 150 mM NaCl, 1% NP40, 0.5% sodium deoxycholate, and 0.1% sodium dodecyl sulfate [SDS]), added with protease inhibitors (pestatin, leupeptin, aprotinin, quimostatin, antipain, and PMSF) and phosphatase inhibitors (Na3 VO4, and NAF), and clarified by centrifugation at 4°C for 20 min. Protein concentration was determined by the Lowry method (DC Protein Assay, Bio-Rad). Forty micrograms of total protein were mixed with loading buffer, resolved on a 7.5-12% SDS- polyacrylamide gels under denaturing conditions and electro-transferred to polyvinylidene difluoride membranes (Bio-Rad, CA). Nonspecific binding was blocked with 5% milk and 1% bovine serum albumin solution. Then, membranes were incubated at 4°C overnight with primary antibodies (diluted 1:1000 for PRLR (H-300 Santa Cruz Biotechnology), 1:500 for PRL (E-9 Santa Cruz Biotechnology) and 1:10000 for Actin (MAB1501 Millipore), HRP-conjugated anti-rabbit or anti-mouse secondary antibody (Santa Cruz Biotechnology) (diluted 1:5000) was used to reveal the immune detection and blots were developed with a chemiluminescence system (Immobilion, Millipore). As an internal control to confirm that similar amounts of protein were loaded for each lane, actin levels were determined. Optical density measurements were determined and analyzed using Image J analysis software (NIH).

### Fluorescent immunocytochemistry

Cells were grown to 80% confluence on 8-well glass chamber slides cover slips (Labtek, Nalgene) in growth medium. Then, cells were fixed with 4% pararaformaldehide for 10 min at -20°C. Slides were blocked for 90 min with bovine serum albumin (BSA) at room temperature, permeabilized with .2% Tween-20 and then incubated overnight at 4°C with primary antibody diluted 1:50. Next, they were incubated with secondary anti-mouse or anti-rabbit antibodies coupled to Alexa Fluor 488 (Invitrogen) diluted 1:1000 for 1 h. Cell nuclei were counterstained using 1 μg/ml of 4′,6-diamidino-2-phenylindole dihydrochloride (DAPI) (Invitrogen). The fluorescent staining pattern was evaluated using an Axio Imager 2 fluorescence microscope (Carl Zeiss, Göttingen, Germany).

### Total RNA isolation and real-time PCR

Total RNA was isolated using TRIzol reagent (Invitrogen, Carlsbad, CA) according to the manufacturer’s instructions, spectrophotometric quantification at 260, 280 and 230 nm was realized using a NanoDrop 1000 Spectrophotometer (Thermo Fisher Scientific, Inc.). Retrotranscription using 1 μg of total RNA was achieved using M-MLV reverse transcriptase and random primers following the recommended Invitrogen protocol. The real-time PCR reaction was performed using an LightCycler Nano Real-Time PCR System (Roche Diagnostics) starting with a 10 min incubation at 95°C followed by 40 cycles (95°C for 15 sec and 60°C for 1 min). The PCR reaction mixture 20 μl consisted of: 2 μl of cDNA template (100 ng), 1 μl of 20X TaqMan probes (Applied Biosystems) PRLR (Hs01061477_m1 FAM labeled) or PRL (Hs01062137_m1 FAM labeled) specific, 10 μl of 2X TaqMan Gene Expression Master Mix (Applied Biosystems) and 7 μl of RNase-free water. PRL and PRLR mRNA relative expression was calculated using the 2- ΔΔCq method after validating similar reaction efficiencies of both the interest gene (PRL and PRLR) and the reference gene 18s RNA (Hs03928985_g1 VIC labeled, Applied Biosystems) by running serial dilutions of both genes [30].

### Cell proliferation assay (MTTAssay)

Cells (5 × 10^3^) were seeded in 96-well plates and were allowed to grow for 24 h in growth medium. Growth medium was replaced with serum free medium supplemented with 10% charcoal striped serum (CSS) before dosing with PRL 200ng/ml (L7009 Sigma), PRLR neutralizing antibody (MAB1167 R&D systems) or PRL antibody (6F11 QED Bioscience) for 3 or 5 days. Then 5 μl of 3-(4,5-dimetylthiazol-2-yl)-2,5-diphenyl-tetrazolium reagent (MTT) (5mg/ml) was added to cells and the cultures and incubated for 3 h at 37°C in a CO2 chamber. Blue formazan crystals were solubilized with acidified isopropanol, and formazan levels were determined by measuring absorbance at 570 nm in an Epoch Microplate Spectrophotometer (Bio-Tek Instruments, Inc.).

### Apoptotic assay (TUNEL Assay)

For the TUNEL Assay we used the kit APO-BrdU (Invitrogen). Cells were grown for 24 h in 8-well chamber slides seeded with 5 × 10^4^ cells per well were treated and incubated with etoposide alone and in the presence of PRL or PRL antibody for 24 hours at 37°C. The slides were washed in PBS and fixed with 4% paraformaldehyde for 30 min at room temperature. Fixed cells were washed in PBS, permeabilized with .2% Tween-20 for 10 min on ice, and then incubated with terminal deoxynucleotidyl transferase and BrdUTP for 1 h at 37°C. After rinsing with PBS, slides were treated with Alexa Fluor 488 dye–labeled anti-BrdU antibody at 37°C for 30 min and mounted with a glass coverslip. Staining of DNA fragmentation was observed under ultraviolet fluorescent microscope (Carl Zeiss) counting at least 200 cells/well.

### Statistical analysis

Data was analyzed using Graph pad PRISM software (Graph pad version 5.01). Significant effects were determined using ANOVA followed by Student’s t-test, including Dunnett´s post-test for multiple comparisons against a single control group. A statistically significant difference was considered to be present at p<0.05.

## Results

### PRLR is highly expressed in cervical cancer cells

**Figure 1:**
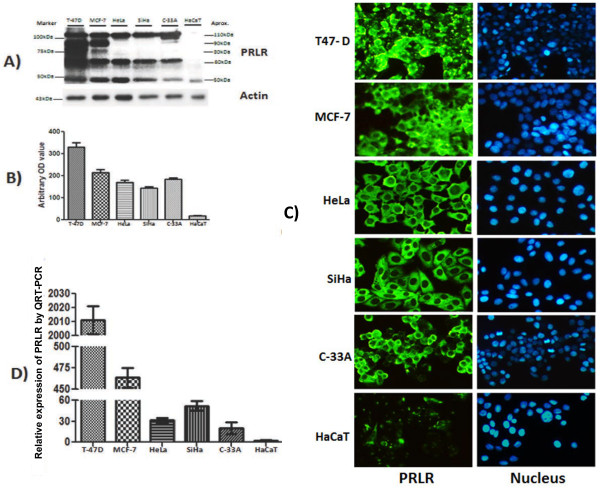
**PRLR expression in human cervical cancer cells.** SiHa, C-33A, HeLa (Cervical cancer cells) and control cells MCF-7, T-47D (breast cancer), HaCaT (Inmortalized human keratinocytes) were cultured in DMEM or RPMI medium containing 10% FBS. **A)** PRLR protein was determined by western blot using a specific antibody against the PRLR, PRLR proteins were identified by their size. **B)** Demonstration of the arbitrary optical density measurements from Western immunoblots assessing PRLR levels. **C)** The cells grown on coverslips were fixed, and the localization of PRLR (green) was observed by inmunocitochemistry using a secondary antibody conjugated with Alexa fluor 488 and DAPI stain (blue) to visualize the presence of cells. Magnification 10 x. **D)** Relative expression of PRLR mRNA was measure by quantitative RT-PCR.

PRLR expression was assessed in cervical cancer cell lines (HeLa, SiHa and C-33A) and human non-tumorigenic keratinocytes (HaCaT) by western blot, immunocitochemistry and real time-PCR. Breast cancer cell lines T-47D and MCF-7 served as the positive control. To detect PRLR expression we used an antibody raised against amino acids 323–622 of human PRLR that recognized multiple isoforms. In T-47D and MCF-7 we observed a high expression of different bands (110 kDa, 90 kDa, 80 kDa, 60 kDa and 50 kDa) that corresponded to PRLR isoforms. Differently than breast cancer cells, in cervical cancer cell lines HeLa, SiHa and C-33A we detected three of the PRLR forms (110 kDa, 60 kDa and 50 kDa). However, human non-tumorigenic keratinocytes (HaCaT) only expressed low levels of the 50 kDa PRLR band (Figure 1A). The optical density measurements from immunoblots demonstrated higher PRLR expression in cancer cell lines in comparison with the non-tumorigenic HaCaT cell line (Figure 1B).

Next, we observed the localization of PRLR by immunofluorescence. The intensity of fluorescence was augmented in cancer cell lines compared with HaCaT, which correlates with western blot results. PRLR staining was heterogeneous on the cell surface and in the cytoplasm of all cancer cells lines (Figure 1C). To evaluate relative mRNA expression levels of PRLR we did real time PCR using a specific probe that could detect all PRLR isoforms. As can be seen, the PRLR mRNA detectable in all cervical cancer cell lines was augmented in comparison with HaCaT that expressed about 15 to 60 fold decrease (Figure 1D). Cell lines T47-D and MCF-7 showed the highest PRLR expression.

### Autocrine Prolactin is produced in cervical cancer cells

**Figure 2:**
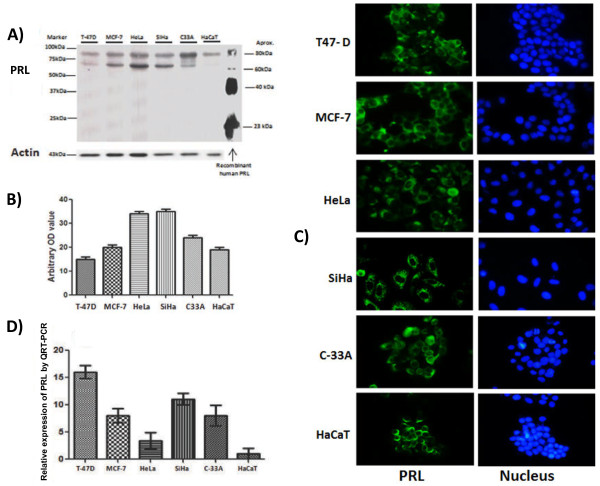
**Presence of autocrine PRL in human cervical cancer cell lines.** SiHa, C-33A, HeLa (Cervical cancer cells) and control cells MCF-7, T-47D (breast cancer), HaCaT (Inmortalized human keratinocytes) were cultured in DMEM or RPMI medium containing 10% FBS. **A)** PRL protein was determined by western blot using a specific antibody against PRL. **B)** Demonstration of the arbitrary optical density measurements from Western immunoblots assessing PRL levels. **C)** The cells grown on coverslips were fixed, and the localization of PRL (green) was observed by inmunocitochemistry using a secondary antibody conjugated with Alexa fluor 488 and DAPI stain (blue) to visualize the presence of cells. Magnification 40 x. **D)** Relative expression of PRL mRNA was measure by quantitative RT-PCR.

PRL expression was detected using the same techniques to evaluate PRLR expression. Our results reveal that cervical, breast cancer and HaCaT cells express PRL-like proteins. Interestingly, PRL found in all cell lines had a higher molecular weight about 60–80 kDa (Figure 2A). The optical density measurements from western immunoblots showed augmented PRL expression in cervical cancer cell lines in comparison with the non-tumorigenic HaCaT cell line (Figure 2B). A similar localization of PRL was observed in all cells studied, the intensity of fluorescence was augmented in T47-D, MCF-7 and HeLa (Figure 2C). The mRNA levels were increased in cancer cells compared with HaCat (Figure 2D).

### Effects of prolactin and PRL/PRLR blocking antibodies on cell proliferation in cervical cancer cells

**Figure 3:**
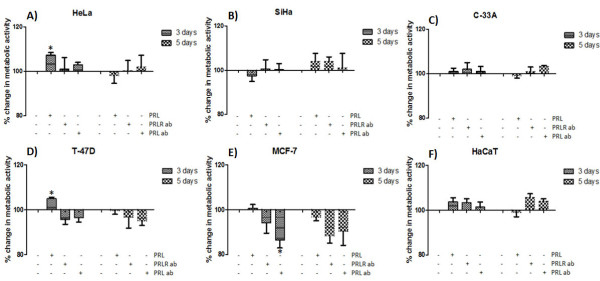
**Effects of PRL and PRL or PRLR blocking antibodies on proliferation of cervical cancer cells.** Effects on metabolic activity after the incubation with PRL (200 ng/ml), PRL-AB (200 ng/ml) or PRLR-AB (2.5 μg) for 3 or 5 days in HeLa, SiHa, C-33A **(A**, **B**, **C)** and control cells MCF-7, T-47D and HaCaT **(D**, **E**, **F)**. Graphs show experiments performed in triplicate, which are repeated at least three times. *p<.05.

To test whether PRL had an effect on the proliferation in cervical cancer cells, we cultured cells in the absence and presence of PRL, PRLR-AB or PRL-AB for 3 and 5 days. Treatment with PRL (200 ng/ml) increased the viable cell number in HeLa about 9% after 3 days of incubation (Figure 3A); while no effect was observed in SiHa and C-33A (Figure 3B, C). The incubation with PRLR-AB (2.5 μg) or PRL-AB (200 ng/ml) had not impact over the total cell number in cervical cancer cells. Similar dose response analyzes with PRL and blocking antibodies were performed in breast cancer and HaCat cells, observing an increase of 6% in the cell number of T47-D after 3 days of incubation with PRL. Moreover, the treatment with PRL and PRLR antibodies decreased the total cell number in a range of 5–8% in T47-D and 12-18% in MCF-7 at day 3 (Figure 3D, E). But, this decrease was statistically significant only in MCF-7 after 3 days of incubation with the PRL-AB. No effect on cell proliferation in HaCat cells was observed (Figure 3F). Other dose and time conditions were used in all the cell lines and no significant difference was detected in the total cell number (data not shown).

### Prolactin treatment reduce DNA fragmentation induced by etoposide

**Figure 4:**
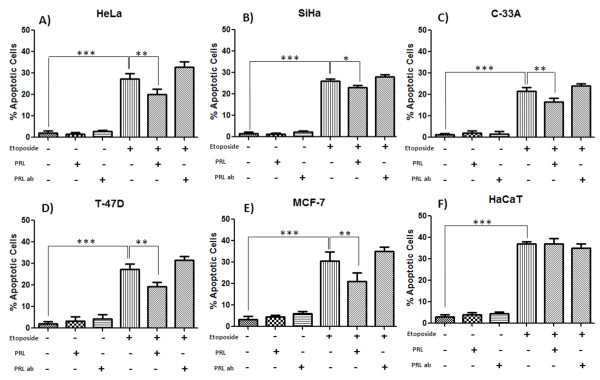
**Effects of PRL and PRL antibody on apoptosis induced by etoposide.** Effects of the treatment with etoposide (40 ug/ml) for 24 hrs with or without added PRL (200 ng/ml) or PRL-AB (200 ng/ml) in SiHa, C-33 A, HeLa **(A**, **B**, **C)** and control cells MCF-7, T-47D and HaCaT **(D**, **E**, **F)**. DNA strand breaks were analyzed microscopically by TUNEL. Graphs represent the mean of at least three replicate, where *p<0.05, **p<0.01 and ***p<.001.

Treatment with etoposide (1 μM) augmented the number of cells with fragmented DNA in all cell lines after 24 h of incubation, as expected. PRL (200 ng/ml) co-stimulus significantly decreased the number of cells with DNA fragmentation from 27.3 to 19.1% in HeLa cells, from 26.7 to 21.2% in SiHa cells and from 22.1 to 16.4% in C-33A (Figure 4A, B, C). Also, PRL treatment decreased induced cell death in breast cancer cells, from 28.7 to 18.3% in T47-D cells and from 30.6 to 21% in MCF-7 cells (Figure 4D, E). PRL had no effect on cell survival in HaCat cells pretreated with etoposide (Figure 4F). PRL-AB treatment (200 ng/ml) did not modify apoptosis induced by etoposide in any of the cell lines tested. Also, no effect on DNA fragmentation was found when PRL-AB or PRL was used.

## Discussion

To date, several reports associate the expression of the PRL/PRLR with the development and progression of cancers such as breast, prostate, colorectal, gynecological, laryngeal, and hepatocellular [31]. There are few reports that have focused on the analysis of the PRL or PRLR expression and its possible role in cervical cancer remains unknown. A previous study demonstrates that the presence of PRL was augmented in malignant cervix tissues [28]. In another work, they reported an increment of serum PRL levels in a considerable number of patients with cervical cancer [29]. In a recent study of our investigation group we observed an increased PRLR expression in cervical cancer samples compared with intraepithelial cervical lesions (data not published). With this background information, we decided to evaluate the expression levels of PRL and PRLR and their possible participation in cell survival of cervical cancer cell lines.

The results of this study demonstrate that PRLR are over-expressed at protein and mRNA levels in human cervical cancer cells compared with human non-tumorigenic keratinocytes. Heterogeneous PRLR staining was observed on the cell surface and in the cytoplasm. Previous reports have proposed a mechanism by which PRLR might become stabilized and accumulated in breast cancers. The proposed mechanism states a constitutive oncogenic signaling downstream of the Ras pathway which inactivates Glycogen synthase kinase 3 beta and prevents phosphorylation of PRLR on Ser349 and PRLR ubiquitination, ultimately leading to PRLR stabilization [32,33].

Moreover, when we evaluated PRLR expression in protein extracts from the cervical cancer cell lines, we observed a high expression of 110 kDa, 60 kDa and 50 kDa bands that could correspond to different PRLR variants previously reported [17-19]. While, in HaCaT cells, only a 50 kDa band was detected. Actually, several investigators have focused in revealing the impact of the expression of specific PRLR isoforms. They have described that short PRLR forms act as dominant negative regulators of the stimulatory actions of the long PRLR forms in vitro. In prostate cancer, it has also been demonstrated that the long term increased expression of the PRLR short form 1b in PC-3 human prostate cancer cells decreases cell growth and migration, and causes multiple changes in gene expression consistent with reduced invasive capacity [34]. Another report showed a low ratio of short to long PRLR forms in breast cancer tumors when compared with normal samples; this reduced expression in patients with cancer could contribute to breast tumor development and progression [20]. The expression of multiple PRLR products (that might correspond to long and short PRLR forms) observed in cervical cancer cells lines suggests that the specific evaluation of the PRLR isoforms could be important for the diagnosis or treatment of the disease.

Furthermore, we showed an autocrine PRL synthesis in cervical cancer cells through the PRL transcript and protein. PRL found in the cell lysates had a higher molecular weight (60–80 kDa) than the PRL peptide of 23 kDa commonly reported. This data is consistent with a previous report that demonstrated the presence of a 60 kDa PRL-like variant in both normal and systemic lupus erythematosus (SLE) PBMNC extracts, preferentially expressed in SLE subjects [35]. This PRL variant could be generated by post-translational modifications such as glycosylation or phosphorylation [36,37].

PRL and PRLR co-expression observed in cervical cancer cells suggests the existence of an autocrine–paracrine loop of action supporting the cell growth in cervical cancer. The results of this study showed that PRL treatment had significant effects only on HeLa cell proliferation, yet these effects were not observed in C-33A or SiHa. This observed difference in response between cervical cancer cells could be due to the origin of the cells. Since SiHa and C-33A are derived from a cervical squamous cell carcinoma, HeLa cell line is derived from cervical adenocarcinoma. Cervical adenocarcinoma arises within glands located in the endocervix, and it is well documented that this kind of tumor has an amplified hormonal response.

Although the major association of PRL with human cancer is given in breast cancer [6,38]; the role of PRL in the proliferation of classical breast cancer cell lines is controversial. Some authors have reported that PRL promotes cell proliferation in some breast cancer cell lines [39]. In contrast, other investigators showed that PRL had no effect on cell proliferation in human breast cancer cell lines MDA-MB-231, T-47D, MCF-7 and Hs578T [40]. The disparity in the results can be influenced by the use of different culture techniques and conditions including the use of different clones of each cell line. One more study, also reported that PRL treatment had no effect on proliferation of LNCaP and PC3 prostate cancer cell lines but showed that PRL has a pro-apoptotic effect in the androgen responsive cell line LNCaP [41].

Several studies have demonstrated that breast cancer and others cell lines like ovarian cancer cells produce autocrine PRL, and that the incubation with PRL antibodies and other PRLR antagonists reduce the cell number [42-44]. In our experiments, we did not find an effect over the proliferation of the cervical cancer cells using PRL or PRLR antibodies, yet a decrease in the number of cells after treatment can be appreciated in the MCF-7 and T47-D cell lines previously reported.

However, we did find that treatment with PRL had a protective effect against cell death induced by etoposide, decreasing the number of apoptotic cells in all the cervical cancer cell lines. Similar results were obtained in T-47D and MCF-7, but not in HaCaT cells. This suggests that PRL has an important role in the cell survival of cervical and breast cancer. A similar result has been reported in breast cancer cell lines T-47D, MCF-7 and Hs578T, showing that PRL has the ability to prevent breast cancer cells from undergoing apoptosis after the treatment with C2-ceramide [40].

At signaling level, it has been reported that even if Jak2 is essential for the proliferative effects of PRL in the onset of induced mammary tumorigenesis, the deletion of Jak2 following neoplastic transformation had no significant impact on the survival and growth of mammary cancer cells in culture and in vivo [45]. We cannot exclude that PRLR signaling is still capable of promoting breast cancer progression and invasion through Jak2/Stat5-independent pathways such as c-Src, FAK, and MAP kinases and beyond towards proliferative effects; thus, other PLR roles are currently being diligently investigated. PRL could favor cell motility and confer resistance to chemotherapy, and thereby contribute to metastasis dissemination [6,46]; such diverse effects may be mediated by distinct PRLR signaling cascades.

## Conclusions

In summary, we demonstrated that human cervical cancer cell lines HeLa, SiHa and C33A over-expressed multiple PRLR forms, and also produced autocrine PRL-like proteins. In addition, PRL augmented cell proliferation in HeLa cells and had a protective effect against etoposide induced apoptosis in HeLa, SiHa and C-33A, suggesting that PRL/PRLR signaling could act as an important survival factor for cervical cancer. Our data supports the hypothesis that the use of an effective PRL antagonist may provide a better therapeutic intervention in cervical cancer. More studies are necessary to determine the signaling pathways activated by PRL and could support the transformation mechanisms activated in those cell lines and hence in cervical cancer.

## Competing interests

The authors declare that they have no competing interests.

## Authors’ contributions

ELP performed all the experimental work described in the study he searched for scientific literature, and contributed with figures. LFJS and MBT contributed with apoptosis experiments and Real Time PCR. JFMV and CEC contributed with scientific ideas and research. STA participated in the study design and contributed to the review of the manuscript. APS conceived and designed the theoretical framework of the study as well as provided scientific guidance throughout the project and wrote the manuscript. All authors read and approved the final manuscript.
